# Estimating the costs and quality of life impact of vision loss in the population aged 50-80 years in Malta: evidence from The Malta Eye Study

**DOI:** 10.3389/fpubh.2025.1706208

**Published:** 2025-12-05

**Authors:** David Agius, Julian Mamo, Neville Calleja, Andrew F. Smith, Francis Carbonaro

**Affiliations:** 1 Department of Surgery, University of Malta, Msida, Malta; 2 Department of Ophthalmology, Mater Dei Hospital, Msida, Malta; 3 Department of Public Health, University of Malta, Msida, Malta; 4 MedMetrics Inc., Ottawa, ON, Canada; 5 Department of Twin Research, King’s College, London, United Kingdom

**Keywords:** visual impairment, ocular disease, economic burden, cost analysis, productivity losses, cataract, refractive error, visual disability

## Abstract

**Background:**

Visual impairment and related ocular conditions impose substantial direct, indirect, and intangible costs, encompassing healthcare expenses, productivity losses, and reduced quality of life. Despite the global relevance of visual impairment, no comprehensive cost analysis has yet been conducted in an older adult Maltese population aged 50–80 years.

**Methods:**

Prevalence estimates from the population-based Malta Eye Study were used to calculate indirect costs via the gross national income per capita method with disability weight assumptions. Direct medical costs for key ocular conditions, including refractive error, cataract, age-related macular degeneration, diabetic retinopathy, and glaucoma, were estimated using prevalence, hospital, and private data performance indices and relevant cost data, enabling estimation of service coverage and unmet care needs. Intangible costs were derived from quality-of-life measures using the National Eye Institute Visual Function Questionnaire–39 to calculate disability weights and years lived with disability (YLD).

**Results:**

The productivity losses from blindness and moderate–severe visual impairment among individuals aged 50–80 were estimated at €16.0 million per annum (95% CI €6.0–€43.0 million). The estimated annual direct medical costs from the main ocular causes were estimated to sum up to €53.4 million (95% CI €44.6–€67.0 million), with unmet needs amounting to €20.8 million (95% CI €15.5–€28.5 million). Cataract (56.9%) and refractive error (24.5%) accounted for the highest shares of such costs. Vision-related quality of life correlated with the severity and laterality of visual impairment. Mild unilateral visual impairment carried the highest YLD rate 2264.4 YLDs per 100,000 while uncorrected refractive error carried the highest YLD rate among the visually impairing causes (2452.7 YLDs per 100,000).

**Discussion:**

Visual impairment imposes a considerable economic and quality-of-life burden on an older adult population in Malta, driven largely by cataracts, refractive error, and productivity losses. These results emphasize the need for preventive and treatment strategies and underscore the importance of future cost–benefit and cost-effectiveness analyses to help guide eye health policy in Malta.

## Background

In 2020, among people aged 50 years and above, the global age-standardized prevalence rates were estimated at 1.85% for blindness, 11.18% for moderate-to-severe visual impairment (MSVI), 7.73% for mild visual impairment, and 22.33% for uncorrected presbyopia (1). Visual impairment (VI) and associated ocular conditions impose a substantial economic and societal burden, which can be broadly classified into indirect, direct, and intangible costs. Indirect costs arise from productivity losses among affected individuals and their informal carers. Direct medical costs include expenses for screening, diagnosis, treatment, and follow-up care, while direct non-medical costs include informal caregiving, transportation, assistive devices, housing adaptations, and all other non-medical costs. Intangible costs reflect reductions in quality of life and healthy life years (2–7).

Despite the global relevance of VI, comprehensive cost data are limited, and no previous cost-of-illness study has quantified this burden in the Maltese population, creating a critical evidence gap in understanding its financial and societal implications. The GDP per capita has increased dramatically in recent years, with 2024 rates at 109% of the European mean (8), and the VI impact remains economically relevant and rising. Conversely, several European studies have estimated the substantial economic burden associated with VI. For example, in the United Kingdom, the economic cost of sight loss and blindness was estimated at approximately £15.8 billion in 2013 (6). In Germany, non-medical annual costs per affected individual were estimated at €12,662, and in the UK, €13,674 (9), while a Spanish societal-perspective model projected a cumulative cost of €99.8 billion between 2021 and 2030 for major vision-impairing diseases. This has reported annual per-patient costs of €22,251 for glaucoma, €13,855 for diabetic retinopathy (DR), €12,706 for diabetic macular edema (DME), €10,634 for age-related macular degeneration (ARMD), and €9,909 for myopic macular degeneration (MMD), with projected increases of approximately €1,000 per condition by 2030 (7). These findings highlight the magnitude of VI-related costs across Europe and the importance of generating country-specific data to inform national health policy and resource allocation. As Malta’s economy is undergoing rapid growth, the impact of VI will grow, accompanied by the need for resources to tackle it.

Estimating indirect costs typically involves applying disability weights to prevalence data, assuming complete productivity loss for blindness and partial loss for MSVI, alongside additional caregiver productivity reductions (2, 4). In Malta, previous European estimates extrapolated regional prevalence data and applied local economic indicators, suggesting productivity losses ranging from €10 to €20 million, depending on the model used (10). Social security benefits related to VI in Malta amounted to €2.86 million in 2024 (as per previous communication with G. Cremona, 2025).

Direct medical costs are variable, reflecting differences in disease severity and treatment intensity. Prevalence-based cost analyses, often adjusted for purchasing power parity (PPP), provide a practical approach for estimating annual costs at the population level. In high-income countries, for example, cataract surgery costs exceed $2,500 per episode, spectacles cost ~$201, DR management with anti-VEGF costs ~$40,825 over five years, and glaucoma and ARMD treatments cost ~$5,272 and ~$4,824 annually, respectively (3). Many cases of VI due to cataract or uncorrected refractive error are avoidable (11), and interventions such as cataract surgery (12), refraction, and corrective lenses (13). They are highly cost-effective, reducing both individual and healthcare system burdens.

Finally, intangible costs capture the broader impact of VI on quality of life. Disability weights derived from the Global Burden of Disease (GBD) study indicate that VI and blindness rank 9th globally in terms of years lived with disability (YLDs), accounting for 29.2 million YLDs and an age-standardized rate of 342.8 per 100,000 population (14).

The Malta Eye Study (TMES) is a population-based investigation of VI and ocular disease in adults aged 50–80 years (15, 16). By combining TMES epidemiological data with local healthcare and social security cost information, this paper addresses this gap. It focuses on the direct, indirect, and intangible costs of VI, as well as its main causes in the older adult population of Malta.

## Methods

The cost analysis incorporated data collected from TMES as part of a prevalence study, which were then applied to cost data from other sources, as detailed below.

### Prevalence study

The Malta Eye Study (TMES) is a population-based cross-sectional survey of adults aged 50–80 years in Malta. The detailed methodology of TMES as a prevalence study has been reported elsewhere (15). A random sample (*n =* 4,006) was selected from the national electoral register, yielding a representative sample of 1,794 individuals who attended the ophthalmic assessment between September 2021 and July 2024 in Mater Dei Hospital and Gozo General Hospital.

VI was defined according to ICD-11 criteria, using distance-presenting visual acuity. Moderate-to-severe VI (MSVI) was defined as a presenting visual acuity of >0.5–1.3 logMAR in the better-seeing eye, while blindness was defined as >1.3 logMAR.

### Ethical clearance and permissions

Ethical approval was granted by the University of Malta Faculty Research Ethics Committee (FRECMDS_1819_94) before data collection commenced in 2019. The study followed the Declaration of Helsinki (17) and GDPR (18) guidelines, with hospital and Data Protection Officer approvals.

### Cost analysis

#### Indirect costs

Indirect costs were calculated for VI using the 2024 gross national income (GNI) per capita, following the formula and disability weight assumptions as described by Eckert et al. (2). Since most employed participants were aged 50–64, productivity losses were estimated for this age group, assuming that carers were <65 years. As derived by Eckert et al. (2), blindness was modelled as a total productivity loss plus 10% caregiver loss, while MSVI was modelled as a 30% productivity loss plus a 5% caregiver loss. For those aged 65–80, only caregiver productivity losses were considered. To assess the robustness of indirect cost estimates, a one-way sensitivity analysis was performed by varying productivity losses and caregiver loss assumptions for blindness and MSVI by ±10%, with the boundaries constrained to 0% as the minimum and 100% as the maximum. The ±10% range was selected as a conventional deterministic variation commonly applied in cost-of-illness studies when precise uncertainty ranges are unavailable, providing a reasonable test of the stability of results to moderate parameter changes (19, 20).

#### Direct costs

This cost analysis was conducted primarily from a public health system perspective, focusing on costs borne by the national healthcare provider. When public sector prices were unavailable, private sector estimates (minimum prices) were used, and vice versa, providing a broader estimate of the total economic burden while remaining comparable with public health expenditure frameworks.

Direct medical costs were estimated for five common visually impairing ocular conditions of public health importance in Malta, as determined by their proportion of VI in TMES (21): refractive error, cataract, DR, ARMD, and glaucoma. For each condition, costs were stratified into met and unmet needs. Unmet needs were extrapolated from the prevalence of undiagnosed or unmanaged cases identified in TMES to the national 50–80 population, using recent census data, while met needs were derived from hospital service data (as per personal communication with Borg D., in 2025, Borg C. in 2025 and Portelli E.M. in 2023) or extrapolated TMES findings. Age- and sex-adjusted prevalence estimates were applied to the national population to determine the total number of individuals with each condition, which were then multiplied by unit costs to estimate the national direct cost burden.

Costs were assumed to be standard management with full adherence and follow-up, as no local data were available on non-adherence. Disease severity was incorporated where possible: DR was modeled separately and accounted for DME; glaucoma was costed indirectly through drop use prevalence; and ARMD was divided into wet and dry forms. Unit costs were derived from 2025 government tariffs, parliamentary legislation (22), and private-sector quotations, with the lowest private prices selected to ensure conservative estimates. All costs were expressed in nominal 2025 euros (EUR). Older data from 2023–2024 were not inflation-adjusted, given the short interval and negligible effect, and no adjustment for purchasing power parity was made. As this was a cross-sectional cost-of-illness analysis, no discounting was applied. Uncertainty intervals were provided for prevalence-based cost estimates, but not for service-based costing.

Screening costs were included only for glaucoma and DR, reflecting established Maltese programs within Primary Health. However, the estimates used were based on figures from the St. Vincent de Paul Residence program due to a lack of primary health data. For refractive error, both distance and presbyopic correction were included, with met and unmet needs extrapolated nationally from TMES. Only those with presenting VI (logMAR >0.3 improving to ≤0.3 or >N6 at 40 cm improving to ≤N6 with correction) were considered to have unmet needs. Annual refraction and spectacle purchase were assumed, while refractive surgery was excluded. Unit costs for refraction and spectacles were based on the lowest private-sector prices.

For uncorrected refractive error (URE), direct costs included the cost of spectacles, assuming annual replacement, consistent with local optometric practice and insurance norms. To assess the robustness of this assumption, a one-way sensitivity analysis was performed assuming a two-year replacement cycle.

Cataract costs were stratified into met and unmet needs. Met need corresponded to the number of eyes operated on in 2024 across both public and private sectors, with sector-specific pricing applied. Unmet need was derived from TMES VI eyes with cataracts (where VI due to cataracts was defined as the presence of cataracts with a vision >0.3 logMAR), assuming the same sector distribution as for met needs. Each cataract case was assumed to involve one pre-operative and one post-operative visit, consistent with standard practice for uncomplicated surgery in the Maltese public sector, while biometry and B-scan ultrasonography were included in public sector surgical costs. This reflects typical resource use per patient and aligns with local ophthalmology guidelines. Simultaneous bilateral surgeries and post-operative complications were excluded, and YAG capsulotomy was not included due to the unavailability of clinical performance data and timelines.

For ARMD, costs were calculated separately for non-neovascular and neovascular cases, with wet ARMD assumed to be treated in the public sector with intravitreal bevacizumab. Prevalence was extrapolated from TMES. Management followed a pro re nata regimen with six injections and nine clinic visits annually between years two and seven, in line with Hujanen et al.’s data (23) with OCT imaging accompanying each injection.

DR costs were modeled using TMES questionnaire data on screening and review combined with clinical findings, with separate costing for DME and non-edematous cases. DME patients were assumed to receive an average of 2.9 intravitreal bevacizumab injections annually by year six, based on IRIS registry data (24). OCT scans were assumed to be 2.5 times per year for DME and twice yearly otherwise. Panretinal photocoagulation, fluorescein angiography, steroid injections, and diabetic vitrectomy were excluded due to insufficient data on annual clinical performance. All DR care was assumed to occur in the public sector, with complications not included.

Glaucoma costs incorporated diagnosed and undiagnosed glaucoma, ocular hypertension, and suspects, with met and unmet needs derived from TMES prevalence. Screening was assumed for individuals with a family history but no clinical signs, with no distinction between met and unmet needs due to limited data. Management assumed 1.5 ophthalmology visits and one visual field test annually. OCT imaging, laser trabeculoplasty, YAG iridotomy, and surgery were excluded from annual costing, given TMES’s cross-sectional design and lack of timelines. Newly diagnosed cases were assumed to start once-daily latanoprost.

Additional glaucoma surgeries reported in TMES but with unclear indication or timeline (e.g., vitrectomy, glaucoma surgery) were costed separately using 2024 public sector performance indices.

To explore the robustness of our cost estimates, we conducted a validation exercise by applying annual direct cost figures reported in the literature to the prevalence of visual impairment in our study population. This allowed us to compare externally derived cost estimates with our base-case results and assess their consistency. Discrepancies were interpreted in the light of differences in healthcare organization, cost of living, and service utilization patterns between Malta and other settings.

#### Intangible losses

Intangible losses were quantified from data obtained from TMES. A generalized linear model (GLM) with a Gamma distribution and log link was applied to European Quality of Life EQ-5D-5L (25) utility scores, which were non-normally distributed. Predictors included the National Eye Institute Visual Function Questionnaire – 39 (NEIVFQ) (26) scores, VI categories, causes of visual impairment, age, sex, and comorbid conditions such as hypertension, diabetes, other systemic illnesses, and the Quick Mild Cognitive Impairment Screen (QMCI) (27). All covariates were retained in the model regardless of statistical significance to ensure that predicted EQ-5D-5L values reflected the combined effects of all variables. Adjusted predicted EQ-5D-5L values were then derived for each individual.

Disability weights (DWs) were calculated by subtracting the predicted EQ-5D-5L values from 1, in line with the definition of DWs as the complement of utility scores. These weights thus incorporated the VI status together with comorbidities. For each condition, prevalence was calculated as the number of affected individuals divided by the total study population, and Years-Lived-with-Disability (YLDs) were estimated by multiplying the prevalence of each condition by its average disability weight, following WHO (28) methodology. This was in turn multiplied by the actual population number within the age group in question to deduce the absolute YLD, and multiplied by 100,000 to deduce the YLD rate per 100,000.

## Results

The key prevalence results from TMES are described elsewhere (16). This paper shall focus on the derived costing estimates based on TMES prevalence data and separately obtained clinical performance data and costings.

### Employment

Within the adjusted TMES sample population, the employment rate varied by age group and decreased drastically by the age of 65. The age range of 50–64 years represented 86.7% (95% CI 84.1–89.1%) of employees within the TMES population. Among the adjusted TMES population in employment, 95.7% (93.9, 97.0%) had no VI in the better eye.

### Indirect costs

In TMES, it was found that employment level decreased significantly from the age of 65, and hence the number of visually impaired (blind/MSVI) among the 50–64-year-old age group was considered as the number of visually impaired in the working ages.

Estimated productivity losses from blindness and MSVI among individuals aged 50–80 totaled approximately €16.0 million per annum (95% CI €6.0–43.0 million) (Table 1). Using a one-way sensitivity analysis, varying the disability weights by ±10% around the base values derived from Eckert et al., combined productivity losses ranged from €10.4 million per annum (95% CI 2.6–25.5 million) with lower weights to €27.0 million per annum (95% CI 11.0–64.5 million) with higher weights.

**Table 1:** Estimated productivity losses from blindness and MSVI among individuals aged 50–80 in Malta.

Visual impairment group	Age group of impaired individuals	Individual/carer losses	Gross national income *per capita* calculation			National minimum wage calculation		
			Loss (€)	L 95% CI (€)	U 95% CI (€)	Loss (€)	L 95% CI (€)	U 95% CI (€)
Blindness	50–64	Individual	3,034,539	76,827	16,866,227	1,068,648	27,055	5,939,634
		Carer	303,454	7,683	1,686,623	106,865	2,706	593,963
	65–80	Carer	876,532	180,910	2,552,890	308,681	63,709	899,029
	Total	Total	**4,214,525**	**265,419**	**21,105,740**	**1,484,193**	**93,470**	**7,432,626**
Moderate Severe Visual Impairment	50–64	Individual	8,193,256	3,754,267	15,486,369	2,885,348	1,322,108	5,453,701
		Carer	1,365,543	625,711	2,581,061	480,891	220,351	908,950
	65–80	Carer	2,191,330	1,230,557	3,593,478	771,702	433,354	1,265,484
	Total	Total	**11,750,129**	**5,610,534**	**21,660,908**	**4,137,942**	**1,975,813**	**7,628,135**
Total	Total	Total	**15,964,655**	**5,875,953**	**42,766,648**	**5,622,135**	**2,069,284**	**15,060,761**

The prevalence of blindness (0.1, 95% CI 0.0, 0.6%) and MSVI (1.0, 95% CI 0.4, 1.8%) among 50–64-year-olds obtained from TMES (*n =* 938) was used to extrapolate the number of blind (*n =* 96, 95% CI 2–535) and MSVI (*n =* 866, 95% CI 397–1637) in Malta in the same age group (*n =* 90,237). Similarly, among the individuals aged 65-80, the prevalence of blindness (0.4, 95% CI 0.1–1.0%) and MSVI (1.8, 95% CI 1.0–2.9%) obtained from TMES (*n =* 847) was used to extrapolate the number of blind (*n =* 278, 95% CI 57–810) and MSVI (*n =* 1,390, 95% CI 781–2,2279) in Malta in the same age group (*n =* 78,486). The 2023 Gross National Income, €17.77 billion (29), amounting to €31,531 per capita (30), and the 2023 annual National Minimum Wage of €11,104 (31) were taken into consideration.

The largest contributors were URE and cataract, with smaller contributions from amblyopia, DR, ARMD, glaucoma, and pathological myopia (Supplementary Tables 1, 2).

### Direct costs

The estimated annual direct medical costs, whether met, unmet, or unknown, related to refractive error (Table 2) and cataract (Tables 3, 4). ARMD (Table 5), DR (Tables 6, 7), and glaucoma (Table 8) sum up to €53.4 million (95% CI €44.6–€67.0 million), with met needs amounting to €27.6 million (95% CI €25.8–€30.6 million) and unmet needs amounting to €20.8 million (95% CI €15.5-€28.5million). Cataract accounted for the highest share of total care costs (56.9%), contributing 46.6% of all met need costs and 84.1% of all unmet need costs. Refractive error followed (24.5% of total costs), with a higher contribution to meet need costs (42.8%) than to unmet need costs (5.9%).

**Table 2:** Estimated annual medical costs for distance refractive error and presbyopia correction in Malta, based on TMES prevalence.

Refractive correction status	Type of refractive need addressed	Proportion of patients (%)			Extrapolated population frequency (no. of individuals)	Service required	Annual amount per patient	Price per year (€)		
		Proportion (%)	L95% CI (%)	U95% CI (%)				Price (€)	L95% CI (€)	U95% CI (€)
Met Needs	Met both needs	42.7%	38.7%	46.8%	72,045	Refraction	1	2,521,573	2,285,902	2,761,501
						Glasses	2	4,178,607	3,788,065	4,576,201
	Met one need, no need for others	45.8%	41.8%	49.9%	77,371	Refraction	1	2,707,993	2,469,690	2,948,713
						Glasses	1	2,243,766	2,046,315	2,443,220
	Met one need, unmet the other	3.7%	2.3%	5.5%	6,167	Glasses*	1	178,851	112,766	268,233
	Total	88.5%	80.5%	96.7%	149,416			**11,830,791**	**10,702,738**	**12,997,868**
Unmet Needs	Met one need, unmet the other	3.7%	2.3%	5.5%	6,167	Refraction	1	215,855	136,096	323,729
						Glasses	1	178,851	112,766	268,233
	Unmet one need, no need other	7.8%	5.8%	10.2%	13,176	Refraction	1	461,144	342,147	605,245
						Glasses	1	382,091	283,493	501,489
	Both needs Unmet	0.0%	0.0%	0.6%	0	Refraction	1	0	0	36,083
						Glasses	2	0	0	59,795
	Total	11.5%	8.1%	16.3%	15,995			**1,237,940**	**874,503**	**1,794,575**
Total		100.0%	88.6%	113.0%	139,551			**13,068,731**	**11,577,241**	**14,792,442**

This table presents the estimated annual costs for refractive and spectacle services, stratified by met and unmet needs for two forms of correctable refractive error: distance refractive error and presbyopia, based on the proportions of patients with met and unmet needs for distance refraction and presbyopia in TMES. Unit Prices for refraction (€35) and glasses (€29) were obtained from the lowest price available on checking various private optician shops in 2025. Costings for refraction appointments in the public sector were not made available despite requests made to the state primary health service. *Glasses of Met Need. Values in bold represent totals.

**Table 3:** Calculation of Malta’s direct costs for met needs for cataract services in 2024.

Sector	Population frequency (no. of surgical cases/eyes)	Service category	Medical intervention	Amount per patient	Unit price (€)	Population price (€)
Public Sector	4,848	Diagnosis	Ocular Examination	2	40	395,840
		Treatment	Cataract Surgery	1	2,068	10,232,464
			Refraction	1	35	173,180
		Total				**10,801,484**
Private	1,010	Diagnosis	Ocular Examination	2	50	101,000
			Biometry	1	50	50,500
		Treatment	Cataract Surgery	1	1,882	1,900,820
			Refraction	1	35	35,350
		Total				**2,087,670**
Total	5,958					**12,889,154**

For each cataract case operated, two standard clinic assessments (pre- and post-operative), one biometry, and one refraction session were included. For the public sector, biometry was included in the surgical price. Unit Prices were obtained from the billing department at Mater Dei Hospital (public sector) and from the Price List at St James’ Eye Hospital (private sector) (36). The prices for standard cataract surgery with a monofocal implant were used. Values in bold represent totals.

**Table 4:** Estimated direct costs of unmet needs for cataract services in Malta based on TMES prevalence.

Sector	Service category	Medical intervention	Extrapolated Population Frequency (no. of eyes)	Amount per patient	Unit price (€)	Population price (€)		
						Price (€)	L95% CI (€)	U95% CI (€)
Public Sector	Diagnosis	Routine Eye Exam	6,715	2	40	537,169	420,687	675,388
	Treatment	Cataract Surgery		1	2068	13,885,822	10,874,748	17,458,778
		Refraction		1	35	235,011	184,050	295,482
		YAG Capsulotomy	156	1	58.23	4,546	799	36,556
	Total		6,871			**14,662,549**	**11,480,285**	**18,466,204**
Private	Diagnosis	Routine Eye Exam	1,375	2	50	137,528	76,373	227,959
		Biometry		1	50	68,764	38,186	113,980
	Treatment	Cataract Surgery		1	1882	2,588,281	1,437,331	4,290,198
		Refraction		1	35	24,067	26,730	79,786
		YAG Capsulotomy	32	1	408	13,049	1	167,669
	Total		1,407			**2,831,690**	**1,578,621**	**4,879,591**
Total			8,278			**17,494,239**	**13,058,905**	**23,345,795**

Costs were extrapolated from the proportion of eyes with cataract-related visual impairment (*n =* 86; 2.40%; 95% CI 1.92-2.95%) and posterior capsular opacities (*n =* 2; 0.06%; 95% CI 0.01-0.2%) identified in TMES, out of 3,588 eyes assessed, to the national census population aged 50–80 years (*n =* 337,518 eyes from 168,759 individuals). Costing assumptions reflect the proportion of services delivered by the public sector (83%) and are based on met needs. Out of all eyes, cataract management was costed for an estimated 6,715 eyes (95% CI: 5,259–8,442) and 1,375 eyes (95% CI: 764–2,280) using public and private sector rates, respectively. YAG capsulotomy management was similarly estimated for 156 eyes (95% CI: 14–628) in the public sector and 32 eyes (95% CI: 0–411) in the private sector. For each cataract case operated, two standard clinic assessments (pre- and post-operative), one biometry, and one refraction session were included. For the public sector, biometry was included in the surgical price. Unit Prices were obtained from the billing department at Mater Dei Hospital (public sector) and from the Price List at St James’ Eye Hospital (private sector) (36). The prices for standard cataract surgery with a monofocal implant were used. Values in bold represent totals.

**Table 5:** Estimated annual costs related to age-related macular degeneration in Malta, based on TMES prevalence.

Type of ARMD	Laterality	Estimated population frequency	Medical intervention	Annual amount per patient	Unit price (€)	Annual population price (€)		
						Price (€)	L95% CI (€)	U95% CI (€)
Dry ARMD	Any	10,348	OCT and Assessment	1	86.6	896,097	740,375	1,073,167
			AREDS 2 supplementation (30 capsules)	12	14.25	1,769,430	1,461,941	2,119,072
Wet ARMD	Any	658	OCT and Assessment	9	86.6	513,219	206,524	1,055,241
	Unilateral	470	Intravitreal Bevacizumab	6	200	564,411	183,384	1,314,701
	Bilateral	188		12	200	451,529	54,694	1,628,705
Total	Any	11,006	All			**4,194,686**	**2,646,919**	**7,190,887**

This table presents the extrapolated annual care costs associated with ARMD, stratified by type (wet or dry) and laterality (in the case of intravitreal anti-vascular endothelial growth factor [anti-VEGF] injections), based on prevalence estimates from TMES (Dry ARMD 6.1, 95% CI 5.1-7.3%; Wet ARMD 0.4, 95% CI 0.2-0.8%). The annual number of bevacizumab injections was estimated using figures from a recent meta-analysis, based on the median number of pro re nata injections administered during years 2 to 7 of follow-up (23). The unit costs of assessment, OCT and of the administration of intravitreal bevacizumab were obtained from the billing office of the main hospital. The unit cost of the AREDS 2 supplementation was obtained from an internet search of the commonly used formulations of the market, and the lowest price was considered. Values in bold represent totals.

**Table 6:** Calculation of direct costs for met needs for diabetic retinopathy services in Malta.

Ocular condition	Laterality	Estimated Population Frequency	Medical intervention	Annual amount per patient	Unit price (€)	Annual population price (€)		
						Price (€)	L95% CI (€)	U95% CI (€)
No or Early DR	Any	5,832	Health Centre Screening	1	25	145,806	112,213	186,002
Individuals with DR	Any	11,006	Ophthalmologist visits	4	40	1,760,963	1,464,337	2,096,647
Non-DME maculopathy	Any	2,164	OCT	2	46.6	201,645	128,091	301,595
Diabetic Macular Edema	Any	188	OCT	2.5	46.6	21,918	2,655	79,060
	Unilateral	188	Intravitreal Injection	2.9	200	109,120	13,218	393,604
	Bilateral	0	Intravitreal Injection	5.8	200	0	0	402,115
Total		16,838				**2,239,453**	**1,720,515**	**3,459,023**

The costing for such met needs were dependent on the questionnaire responses to whether they visited the health center (3.5, 95% CI 2.7-4.4%) or ophthalmologist (6.5, 95% CI 5.4-7.8%) and on the examination findings in those who reported that they were checking for DR. DME patients (0.1, 5%CI 0.0-0.4%) were assumed to receive an average of 2.9 intravitreal bevacizumab injections annually by year six, based on IRIS registry data (24). DME, Diabetic Macular Edema. Values in bold represent totals.

**Table 7:** Calculation of direct costs for unmet needs for diabetic retinopathy services in Malta.

Ocular condition	Laterality	Estimated Population Frequency	Medical intervention	Annual amount per patient	Unit price (€)	Annual population price (€)		
						Price (€)	L95% CI (€)	U95% CI (€)
No or Early DR	Any	12,323	Health Centre Screening	1	25	308,075	259,084	363,048
Individuals with DR	Any	0	Ophthalmologist visits	4	40	0	0	55,464
Non-DME maculopathy	Any	658	OCT	2	46.6	61,370	24,696	126,185
Diabetic Macular Oedema	Any	282	OCT	2.5	46.6	32,877	6,783	95,926
	Unilateral	188	Intravitreal Injection	2.9	200	109,120	13,218	393,604
	Bilateral	94	Intravitreal Injection	5.8	200	109,120	2,763	607,201
Total		13,546				620,561	306,543	1,641,428

The costing for such unmet needs were dependent on the examination findings in those with DM who reported that they were not checking for DR, including those with no DR/mild/moderate NPDR (7.3, 95% CI 6.1-8.6%) needing health center screening, those who had severe NPDR /PDR /DME (0.0, 95% CI 0.0-0.2%) needing ophthalmologist visits and those who had maculopathy without DME (0.4, 95% CI 0.2-0.8%). DME patients (0.2 95% CI 0.0-0.5%) were assumed to receive an average of 2.9 intravitreal bevacizumab injections annually by year six, based on IRIS registry data (24).

**Table 8:** Calculation of direct annual costs of unmet needs for glaucoma services in Malta.

Met/unmet need	Medical intervention	Population proportion			Estimated population frequency	Annual amount per patient	Unit price (€)	Annual population price (€)		
		Proportion (%)	L95% CI (%)	U95% CI (%)				Price (€)	L95% CI (€)	U95% CI (€)
Unknown	Glaucoma Screening	16.9%	15.2%	18.7%	28,503	1	25	**712,569**	**640,579**	**789,159**
Met Needs	Visual Field Test	2.1%	1.5%	2.9%	3,575	1	11.65	41,644	29,554	56,934
	Ophthalmologist Review	2.1%	1.5%	2.9%	3,575	1.5	40	214,476	152,207	293,221
	Timolol	1.4%	0.9%	2.1%	2,446	12	1.67	49,013	32,089	71,574
	Latanoprost	1.4%	0.9%	2.1%	2,352	12	1.75	49,386	32,030	72,661
	Brinzolamide	0.9%	0.6%	1.5%	1,599	12	1.58	30,320	17,693	48,407
	Brimonidine	0.3%	0.1%	0.6%	470	12	1.78	10,047	3,264	23,402
	Travoprost	0.2%	0.0%	0.5%	282	12	19.3	65,359	13,484	190,699
	Bimatoprost	0.1%	0.0%	0.3%	94	12	28	31,607	800	175,879
	Azarga®	0.1%	0.0%	0.3%	94	12	17.28	19,506	494	108,542
	Trabeculectomy					40*	2,068	82,720		
	Ahmed Valve®					35*	3,085	107,975		
	Total							**702,054**	**472,310**	**1,232,015**
Unmet Needs	Visual Field Test	9.3%	8.0%	10.7%	15,709	1	11.65	183,015	157,358	211,322
	Ophthalmologist Review					1.5	40	942,567	810,428	1,088,351
	Latanoprost					12	1.75	329,898	283,650	380,923
	Total							**1,455,481**	**1,251,435**	**1,680,596**
Total								**2,870,103**	**2,364,324**	**3,701,769**

Costings were based on the number of individuals with known glaucoma or ocular hypertension from TMES (met needs), the number of glaucoma surgeries performed in the public service (met needs), and those with undiagnosed glaucoma, ocular hypertension, or who were identified as suspects in TMES * Annual amount for trabeculectomy and Ahmed valve for 2024 was within the whole population, not per patient.

A one-way sensitivity analysis varying the spectacle replacement interval from one to two years resulted in annual URE costs of €9.5 million (95% CI €8.4–€10.7 million), compared with €13.1 million (95% CI €11.6–€14.8 million) assuming annual replacement.

The costs for the management of posterior capsular opacification by YAG capsulotomy were not calculated because YAG capsulotomy rates were not provided, as clinical performance indices from the hospital clinics do not account for the number of YAG laser capsulotomies done in any given laser clinic. In TMES, 15 eyes out of 3,588 (0.4%; 95% CI; 0.2–0.7%) had evidence of a prior YAG capsulotomy. Extrapolation on the total population’s eyes (*n =* 337,518 eyes from 168,759 individuals) at a 2025 hospital unit price of €58.23 would imply a cost of €1,411 (95% CI €790–€2,324). Since no data were available on the timing of the previous YAG capsulotomy, no annual cost could be calculated.

For glaucoma, the topical treatment cost based on the reported met needs of glaucoma and ocular hypertension, based on the reported prevalence of use of timolol (1.4%; 95% CI 0.9–2.1%), latanoprost (1.4%; 95% CI 0.9–2.1%), brinzolamide (0.9%; 95% CI 0.6–1.5%), and brimonidine (0.3%; 95% CI 0.1–0.6%) amounted to €128,720 (95% CI €81,812-€192,642).

The actual *Pharmacy of Your Choice* individuals registered under glaucoma totaled 9,320 (as per personal communication with Caruana S., 2025). Assuming every individual uses about 1.5 medication bottles per month at an average price of €1.70, this would total €285,192.

Likewise, the proportions of annual panretinal photocoagulation and vitrectomies attributable to diabetic retinopathy remained unknown from both the TMES data and the clinical performance statistics. However, a total of 368 pars plana vitrectomies were performed in the public service in 2024 for various indications, incurring an estimated cost of €1,135,280.

#### Comparative analysis

The mean annual direct medical cost of refractive error correction in our study was €77 per person per year. This estimate is consistent with findings across five European countries in 2009, reporting an average of €145 expenditure every two years, corresponding to €72.5 per year among adults aged ≥45 years (32).

The estimated cost of cataract surgery was €2,163 per eye, within the European range of €432–€3,412. While above many tariffs, it aligns with the upper end in Germany and remains below the highest in Portugal (33).

The estimated annual direct cost of any ARMD was €381 per person, with €258 for dry ARMD and €2,322 for wet ARMD. Compared with published estimates, our overall cost was lower than most reported figures (€1,169–€3,973 annually). The dry ARMD estimate exceeded some specific component costs (e.g., €147.9–€158.1 for geographic atrophy and drusen) but remained below early-stage AMD estimates (€1,399). The wet ARMD estimate lay between lower component costs (€540.1 for choroidal neovascularization) and higher treatment-intensive or advanced-stage costs (€2,787–€5,799) (34).

No reliable estimate could be made for DR, as data on the number of pan-retinal photocoagulations or diabetic vitrectomies were unavailable, with only the number of laser room appointments recorded.

The estimated annual cost of glaucoma management was €196 per person when considering only known cases or €112 per person if also considering undiagnosed cases, substantially lower than most reported ranges (€572–€2,943.83, with €788.70–€8,368.51 depending on stage and severity). While close to Traverso et al.’s Stage 0 estimate (€153) (35), it was below the costs typically reported for early to advanced stages of disease. Surgical management was estimated at €2,543, comparable to filtration surgery (€2,121) and XEN Gel Stent Surgery (€2,297.99), higher than trabeculectomy (€1,236.44), and markedly lower than more complex combined procedures such as iStent inject® with cataract surgery (€8,368.51) (34).

### Intangible losses

#### Self-perceived quality of life

**Figure 1:**
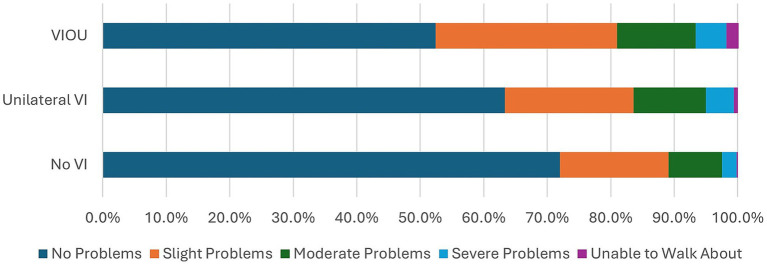
The proportion of severity of mobility dimension from the EQ5D5L by the visual impairment group.

**Figure 2:**
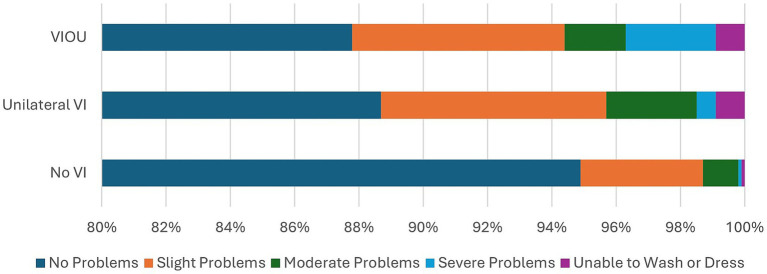
The proportion of severity of the self-care dimension from the EQ5D5L by the visual impairment group.

**Figure 3:**
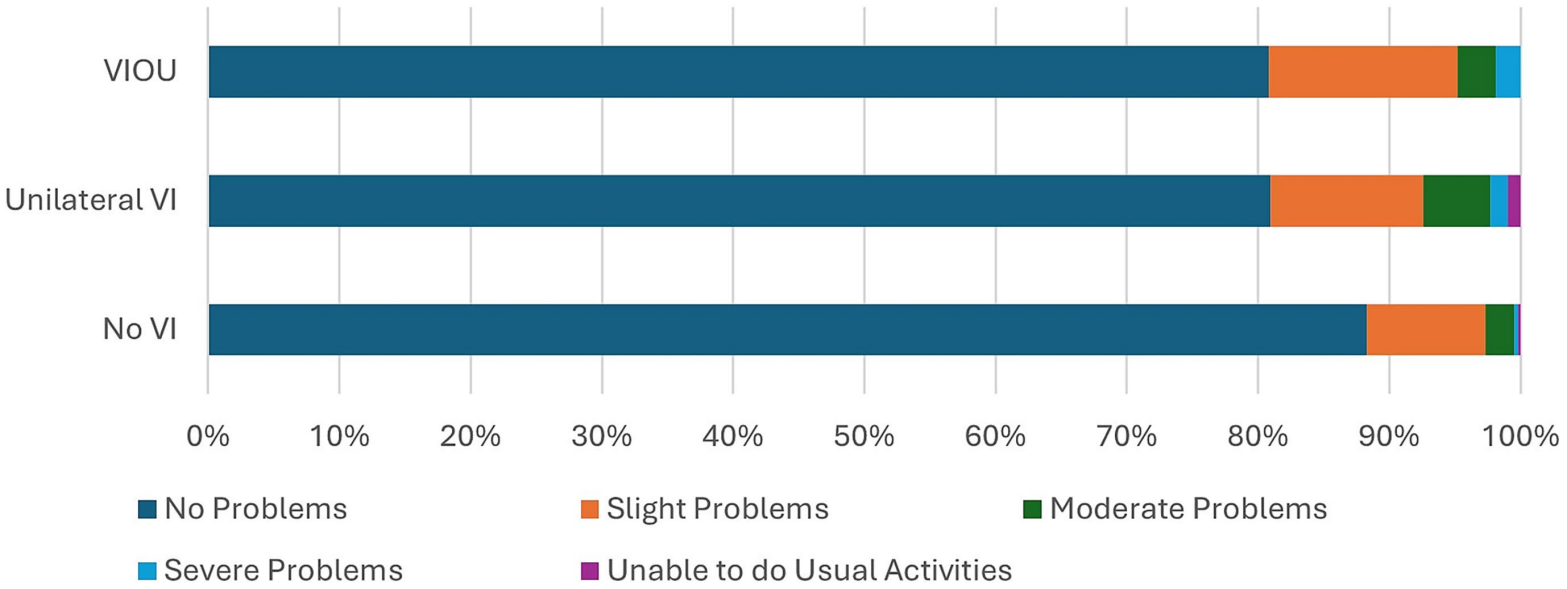
The proportion of severity of the usual activities dimension from the EQ5D5L by the visual impairment group.

**Figure 4:**
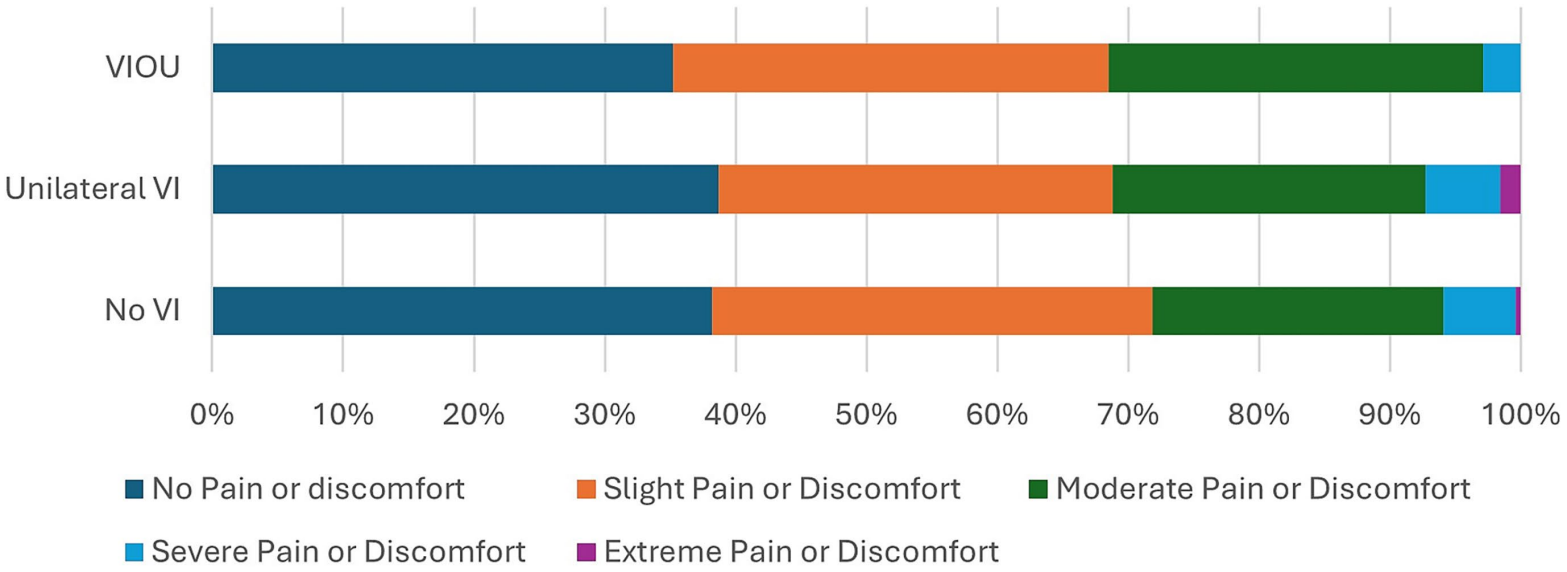
The proportion of severity of the pain or discomfort dimension from the EQ5D5L by visual impairment group.

**Figure 5:**
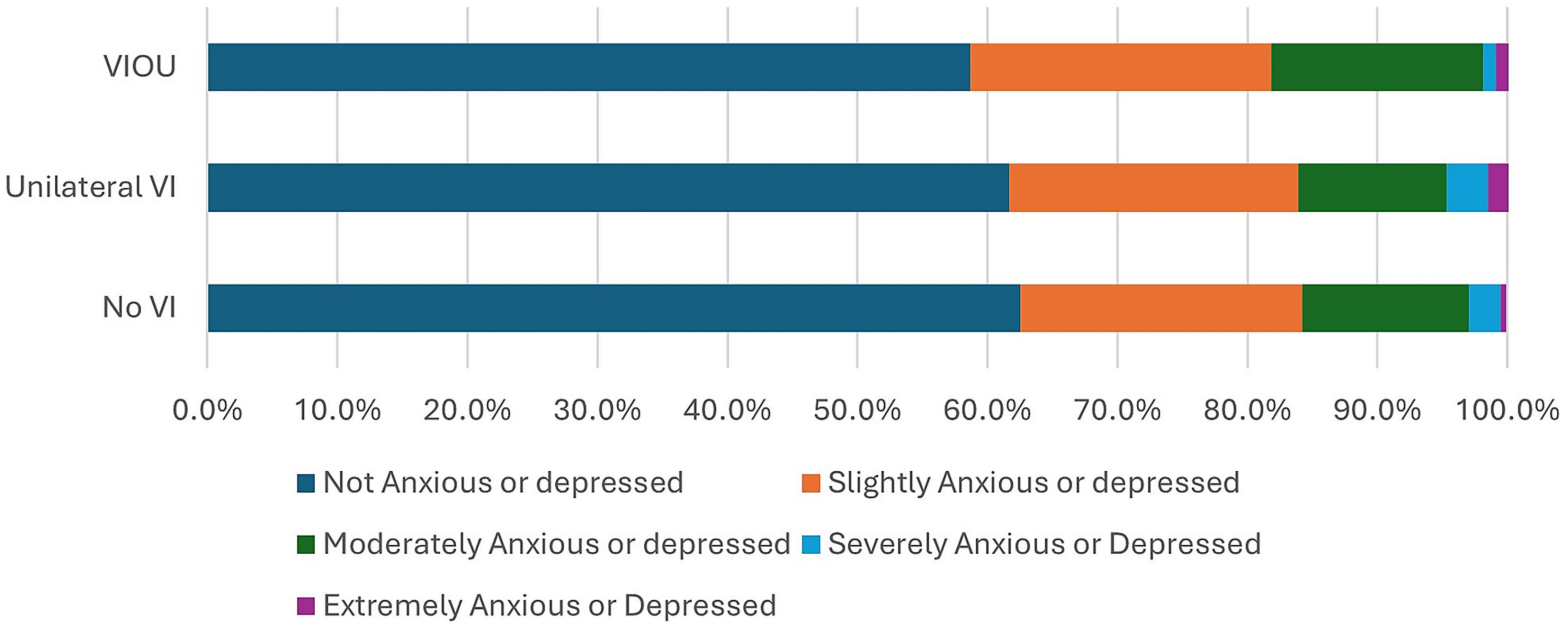
The proportion of severity of the anxiety or depression dimension from the EQ5D5L by visual impairment group.

Comparison of the EQ5D5L questionnaire’s dimensions on quality of life of the no VI group to those of the unilateral VI and bilateral VI (VIOU) groups (Figures 1–5) showed significant differences in proportion of severity of issues with mobility (*p* = 0.001), self-care (p = 0.001), and usual activities (*p* = 0.003), but not for pain or discomfort (*p* = 0.161) and anxiety and depression (*p* = 0.381). Severity of mobility and self-care problems increased with the presence and bilaterality of VI (Figures 1, 2). Issues with usual activities were more prevalent with VI, with the unilateral VI issue group experiencing the highest proportion of severity (Figure 3).

#### Vision-related quality of life

**Figure 6:**
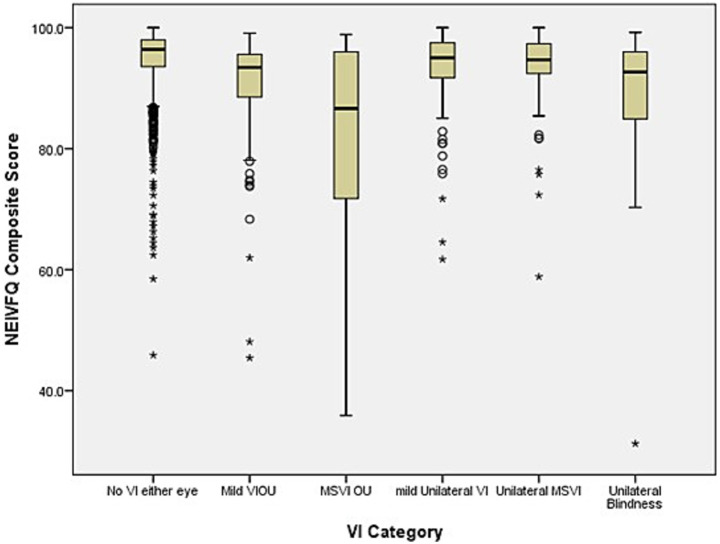
Distribution of the national eye institute visual functional questionnaire (NEI-VFQ) composite scores across visual impairment (VI) groups. Boxplot of NEI-VFQ composite scores by VI status: no VI, mild bilateral VI (mild VIOU), bilateral moderate severe VI (MSVIOU), mild unilateral VI, unilateral moderate–severe VI (MSVI) and unilateral blindness. Individuals with bilateral blindness were excluded from this analysis due to low counts (*n =* 4). The box shows the interquartile range (IQR), with the median represented by a line within the box. Whiskers extend to 1.5 × IQR. Outliers are displayed as circles (°) for values between 1.5 and 3 × IQR from the quartiles, and as asterisks (*) for values beyond 3 × IQR. Differences in distributions were assessed using the Kruskal–Wallis test (*p* < 0.001).

Vision-related quality of life (NEI-VFQ) scores were non-normally distributed in the adjusted TMES population. Participants with VIOU scored significantly lower on the NEI-VFQ composite (Figure 6) and subscale scores than those with unilateral VI, who in turn scored lower than those without VI. Ocular pain scores were not affected. Despite many NEI-VFQ subscales showing median scores near the ceiling (100), Kruskal–Wallis tests identified significant distributional differences in 11 of 12 subscales, particularly near activities, mental health, and social functioning, suggesting meaningful differences masked by similar medians.

#### Years lived with disability

EQ-5D utility scores were positively associated with NEI VFQ-25 scores (B = 0.009, *p* < 0.001), while VI categories and causes of VI were not significantly associated with EQ-5D scores in the gamma regression model. The utility EQ-5D scores were thus adjusted for variables such as age, gender, comorbidities, and the responses from the visual function questionnaire, as well as the severity of visual impairment, even though not all variables were significantly associated with EQ-5D.

The mean predicted EQ-5D score for the study population was 0.80, with individual predicted scores varying according to the severity of VI and other covariates.

While some variables (e.g., severity of visual impairment) showed significant associations with the EQ-5D scores, others (e.g., specific ocular conditions or comorbidities) were not statistically significant. However, all variables, regardless of their significance, were included in the model to adjust the predicted EQ-5D scores and compute the disability weights (DW) for each individual. The proportional DWs, YLD numbers, and YLD rates for each VI condition (severity or cause-related) were calculated accordingly (Table 9).

**Table 9:** Mean disability weights, prevalence, and YLD for VI types, severities, and causes in census-adjusted TMES population.

Visual impairment type/Severity	Mean DW			Prevalence (%)			Absolute YLD number			YLD rate per 100,000		
	DW	L95% CI	U95% CI	Prevalence	L95% CI	U95% CI	YLD	L95% CI	U95% CI	YLD	L95% CI	U95% CI
Mild VIOU	0.21	0.19	0.23	4.5%	3.6%	5.5%	**1564.3**	1112.5	2144.3	**926.9**	659.2	1270.6
MSVI OU	0.20	0.14	0.26	1.3%	0.9%	2.0%	**450.7**	206.5	860.1	**267.1**	122.3	509.6
Blind OU	0.36	0.00	1.12	0.2%	0.1%	0.6%	**135.4**	0.0	1080.9	**80.2**	0.0	640.5
mild Unilateral VI	0.22	0.21	0.23	10.3%	9.0%	11.8%	**3821.4**	3135.6	4613.2	**2264.4**	1858.0	2733.6
Unilateral MSVI	0.22	0.20	0.23	4.9%	4.0%	6.0%	**1795.4**	1323.6	2387.2	**1063.9**	784.3	1414.6
Unilateral Blindness	0.24	0.22	0.27	2.6%	1.9%	3.5%	**1081.6**	708.1	1592.3	**640.9**	419.6	943.5
URE VI	0.20	0.19	0.21	12.3%	10.8%	13.9%	**4139.1**	3438.7	4936.5	**2452.7**	2037.6	2925.2
Amblyopia VI	0.20	0.18	0.22	5.0%	4.1%	6.1%	**1694.1**	1235.3	2273.0	**1003.8**	732.0	1346.9
DR VI	0.27	0.22	0.32	0.8%	0.5%	1.4%	**382.1**	174.4	745.3	**226.4**	103.3	441.6
Glaucoma VI	0.26	0.08	0.44	0.4%	0.2%	0.9%	**195.2**	26.1	648.7	**115.7**	15.5	384.4
Cataract VI	0.27	0.25	0.30	3.8%	3.0%	4.8%	**1743.1**	1234.8	2400.2	**1032.9**	731.7	1422.3
ARMD VI	0.19	0.06	0.33	0.6%	0.3%	1.0%	**182.5**	27.9	563.2	**108.1**	16.5	333.7
MMD VI	0.18	0.13	0.24	1.3%	0.8%	1.9%	**399.0**	183.9	760.5	**236.4**	109.0	450.6

DW: Disability Weight; YLD: Years-Lived-with-Disability. Negative lower confidence bounds for the disability weight of bilateral blindness, resulting from a small sample size, were truncated to 0, as values below zero are not meaningful. Values in bold represent totals.

Out of the VI laterality and severity types, mild unilateral VI carried the highest YLD rate (2264.4 YLDs per 100,000), mainly due to its highest prevalence, while URE carried the highest YLD rate among the VI causes (2452.7 YLDs per 100,000), especially among the 60–69-year-old age group (2735.0 YLDs per 100,000) (Supplementary Table 3), with cataract VI matching that of amblyopia VI, due to a higher DW among individuals with cataract VI.

## Discussion

### Indirect costs and productivity losses

The one-way sensitivity analysis showed that varying disability weights by ±10% resulted in combined productivity losses ranging from €10.4 to €27.0 million per annum, indicating that our indirect cost estimates are reasonably robust. Despite this variation, the economic burden of blindness and MSVI remains substantial, underscoring the importance of interventions to both prevent and/or treat visual impairment.

In estimating indirect costs, the model assumed that individuals aged 65 years or older were no longer active in the labor force, while all those under 65 were working. While this likely excluded a minority who remained economically active beyond retirement age, the model’s estimate of blindness in the better-seeing eye aligned closely with national figures for blindness pension recipients (*n =* 422). This convergence suggested that the assumptions used were broadly reasonable and reflected the true productivity losses attributable to vision impairment within the context of current social support structures.

The use of upper 95% confidence intervals in cases with zero observed frequency, such as bilateral VI from DR, ARMD, or glaucoma in the 50–64 age group, is a conservative approach to account for uncertainty, but it remains a limitation given the absence of directly observed cases.

In Malta, the number of social security beneficiaries for VI as of December 2024 was 422, corresponding well within the extrapolated confidence limits of bilateral blindness (376; 95% CI 103–962), and the total expenditure in 2024 was €2,861,289.47 (as per previous communication with G. Cremona, 2025).

### Direct medical costs

Our results show that the majority of direct medical costs of VI in Malta are due to cataracts, a largely avoidable cause of vision loss. Cataract surgery and refractive error correction are highly cost-effective interventions, highlighting opportunities to reduce both financial and societal burdens. Despite relatively good surgical coverage (16), further prioritization of older adults could decrease prevalence among the most affected age groups. Since late 2024, evening surgical lists have been implemented to reduce prolonged waiting times (37), though age-related declines in health literacy may limit uptake in those >75 years (38).

Limited public-sector optometrists (4/30) may further affect access. Assessing population literacy and willingness on eye checks, the workforce distribution, service structure, and screening effectiveness could guide policies to improve visual outcomes and reduce inequities.

Our estimates generally fall within published European ranges but must be interpreted with caution due to methodological constraints of translating cost structures from other healthcare settings to Malta. In the absence of official public sector pricing, the lowest available private-sector costs were used, which may have underrepresented true expenditures. While a public health perspective was adopted, the mixed cost sources mean estimates may differ slightly from a purely public or private perspective. No inflation adjustment was applied, since the model was based on 2023–2024 pricing. Beyond methodological factors, cost variations across countries also reflect wider economic differences such as standard of living, inflation, economies of scale, and supply-chain or import-related costs, particularly relevant for small island states like Malta, making direct international comparisons inherently difficult. Overlap between disease categories also introduced the possibility of double-counting for individuals with multiple pathologies. Furthermore, it was assumed that any medical costs involving surgery/procedures were not inclusive of complications, thus underestimating costs, while full compliance to treatment was assumed, overestimating real me costs.

For URE, we assumed annual spectacle replacement, which may slightly overestimate actual replacement frequency in the population. Sensitivity analysis assuming a two-year replacement cycle reduced estimated costs from €13.1 million to €9.5 million per annum, indicating that overall conclusions are robust with respect to this assumption. For cataract, it was assumed that participants who had undergone surgery had received at least one biometry and one refraction, though the side (unilateral or bilateral) and exact timing were not known. This may have led to a slight overestimation, particularly if preoperative assessments were duplicated. YAG capsulotomy, a common follow-up intervention, could not be costed due to the absence of data on its use. Cataract surgery costs were notably higher than many international tariffs, which may reflect billing practices in the public sector, where 81% of procedures are performed and only monofocal intraocular lenses are used. Private-sector costs are considerably lower, probably due to differences in consumable amounts and their costs. This reliance on public billing data likely inflated our estimate.

For ARMD and DME the costing model considered all patients with wet ARMD/DME to be ongoing intravitreal therapy at maintenance frequency, as from other published real-world data. This approach did not account for the more intensive injection schedules required in the initial treatment phase and may therefore have underestimated total expenditure. Furthermore, it assumed full access to treatment, though this may not reflect real-world inequalities in uptake or eligibility. Our costing included only bevacizumab, the anti-VEGF agent available in the public healthcare system during the study period. Ranibizumab had not yet been introduced into free public provision, and aflibercept was limited to the private sector, with no utilization data available. While international cost estimates for ARMD are often higher (e.g., Marques et al. (3) ~$4,824 per affected person annually, while our study shows €4,194,686 among 11,006 affected individuals, i.e., €381 per person annually), this may not necessarily indicate an underestimation in our analysis. Rather, it likely reflects genuine cost differences arising from the widespread use of off-label bevacizumab and the more cost-conscious approach to anti-VEGF administration in Malta, especially given the marked cost differences between anti-VEGF agents (39). Similar findings have been reported elsewhere, with bevacizumab achieving comparable visual outcomes to aflibercept and ranibizumab at a fraction of the cost (40). Intensive injection regimens and frequent monitoring schedules remain major cost drivers internationally (41), but these are often optimized in public systems with constrained budgets such as ours. Hence, lower national ARMD costs may accurately reflect the efficiency of local practice rather than an artefactual underestimation. The relatively low rate of ARMD in Malta when compared to European populations (42) further decreases the overall ARMD-related costs per unit population.

In DR, cost modeling was limited by a lack of yearly frequency data for laser treatments such as panretinal photocoagulation or grid laser. Surgical procedures, such as vitrectomy, were also excluded.

The study did not model treatment costs for myopic macular degeneration (MMD), as no data were available to account for the annual intravitreal treatment used for this indication. As MMD is less likely to require ongoing annual treatment compared to ARMD or DME, its exclusion may have had a lesser effect on overall estimates.

The low glaucoma annual cost likely reflects a predominance of early-stage disease in our cohort and the use of inexpensive government-subsidized drops (*Pharmacy of Your Choice*), whereas other studies may include more advanced stages and higher-cost, patient-purchased or preservative-free medications. There is a discrepancy between the calculated government topical treatment cost for glaucoma and ocular hypertension (€285,192) based on the number of *Pharmacy of Your Choice* beneficiaries and the prevalence calculated topical treatment costs (€128,720; 95% CI €81,812-€192,642) and this could be explained by the former including misdiagnosed cases and the assumption that the average glaucoma individual is using 1.5 bottles of treatment per month. Moreover, the costs from this paper were based on TMES data in individuals aged 50–80, while the government figures would have additionally captured younger individuals. A sensitivity analysis with one bottle of treatment per month would have decreased the value to €190,128, and this would correspond better to the prevalence calculated cost. Glaucoma management costs in Malta are substantially lower than reported in most other high-income settings. This likely reflects the predominance of early-stage disease, widespread use of low-cost government-subsidized medications, and limited utilization of complex surgical procedures. The majority of glaucoma cases, however, remain undiagnosed, and the associated per-patient costs are currently minimal, consisting mainly of screening. As a result, the population-level costs are heavily influenced by these unmet needs, which represent latent rather than actual expenditures. If these undiagnosed cases were identified and managed according to standard protocols, total costs would likely rise substantially, highlighting the dual impact of low treatment costs and underdetection. Similar analyses in high-income settings have shown that diagnostic testing and medication costs constitute the main cost components, with the latter being particularly sensitive to local drug pricing and subsidy policies (43).

The introduction of electronic health records is underway, so it is hoped that all this missing data will be available for future studies.

### Direct non-medical costs

Non-medical costs such as travel expenses, time lost from work, expenses related to lifestyle, and home adjustments related to visual disability and informal caregiving were not included in this study. This omission may have resulted in an underestimation of the broader societal cost of VI, particularly for individuals in rural areas, older adults with mobility restrictions, and those undergoing regular follow-up. Patient-reported data would offer a valuable addition to future research in this scarcely explored area.

Annual non-medical costs per person with ARMD have been reported to range between €291.40 and €1745.10, depending on overall vision (44). However, direct extrapolation to Malta is limited, as shorter travel distances, less time lost for hospital visits, and differences in cost of living are likely to reduce the relative weight of non-medical costs compared with larger countries.

### Disability weights and quality of life

Disability weights in TMES were derived from health utility values, which had been adjusted for age, sex, comorbidities, cognitive function (as measured by the Quick Mild Cognitive Impairment Screen), and underlying diagnosis (15). These values were based on EQ-5D and NEI VFQ-25 responses. Compared to global estimates from the GBD framework, TMES reported higher disability weights for mild visual impairment. This difference likely reflected higher functional expectations among participants and the use of a vision-specific quality-of-life instrument.

The estimated YLDs in TMES were broadly similar to those in the GBD study. For moderate to severe visual impairment, TMES reported a YLD burden of 267.1 per 100,000 (95% CI 122.3–509.6), which was within the range of the GBD estimate of 342.8 (95% CI 224.2–503.6) (14). For blindness, TMES estimated 80.2 YLDs per 100,000 (95% CI 0.0–640.5), again showing broad agreement with international estimates, although the wide confidence interval reflected the small number of blind individuals identified in TMES.

### General limitations

Several limitations should be considered when interpreting our findings. First, our analysis is cross-sectional, and the lack of longitudinal data prevents estimation of lifetime costs or changes in visual impairment over time. Second, indirect costs may be underestimated, as we relied on disability weights and productivity loss assumptions without capturing all informal care or societal impacts. Third, national averages were used for key economic parameters, which may not fully reflect local variations in healthcare utilization, costs, or socioeconomic status. Despite these limitations, our study provides the first comprehensive assessment of the direct, indirect, and intangible costs of visual impairment in Malta, offering an important baseline for policy and future research. It is hoped that such additional research will build on what has been collected to date and provide long-term direct and indirect cost collection mechanisms.

### Economic implications of screening and public health policy considerations

While our findings highlight that cataract and URE are the primary drivers of the economic burden of visual impairment in Malta, the cross-sectional nature of the study and the assumptions made in cost modeling limit the ability to directly translate these results into specific public health policy recommendations. Instead, these results identify areas where further research, particularly longitudinal studies, real-world data on spectacle provision, and detailed evaluations of cataract service efficiencies, could inform evidence-based interventions and resource allocation decisions. Moreover, our findings have identified areas where further research, particularly longitudinal studies, real-world data on spectacle provision, and detailed evaluations of cataract service efficiencies, could inform evidence-based interventions and resource allocation decisions.

### Data gaps and future modeling opportunities

More accurate cost modeling would benefit from access to detailed, patient-level data on service use, particularly from longitudinal data from private clinics and outpatient services. The current lack of integrated electronic records for ophthalmic visits, minor procedures, and follow-up appointments limits the precision of estimates. Including variables such as treatment adherence, injection frequency, or complications would further improve modeling accuracy. This could be further remedied by introducing patient cost diaries, which capture direct and indirect expenditures not routinely available in administrative datasets, or by establishing registries that collect more detailed clinical performance data. For example, it is not sufficient to know that laser procedures were performed in outpatient settings; to cost them accurately, one would need to distinguish between YAG capsulotomies, panretinal photocoagulation for proliferative diabetic retinopathy, or focal/grid laser for macular edema. Similarly, identifying how many vitrectomies were performed specifically for diabetic indications would allow a more precise attribution of costs to underlying disease categories. Developing collaborations with private providers to share anonymized service-level data and integrating electronic health records across sectors would further strengthen the accuracy and representativeness of cost estimates. A VI/blindness registry could allow for more accurate longitudinal data on the cost of VI by condition. However, such a registry does not yet exist for the Maltese population. Both the principal investigator (FC) and the lead researcher (DA) are currently collaborating with The Malta Trust Foundation and relevant authorities to establish this registry.

Future analyses should incorporate inflation adjustments and discounting for long-term models and sensitivity testing to evaluate the robustness of key assumptions. Non-medical direct costs should also be included to give a more comprehensive view of the economic burden of vision impairment.

Expanding the costing model for longitudinal data to include cost-effectiveness, cost–benefit, or cost-utility analyses would be useful for justifying interventions such as screening, changes in current practices, and the introduction of future potentially novel eye care interventions and innovations.

Timely access to cataract surgery, particularly for older adults, may reduce indirect costs and disability. The reasons for the high proportion of unmet cataract needs remain unclear; they may relate to poor health literacy, reluctance to undergo ocular surgery, long waiting times for clinic appointments, or extended surgical waiting lists. Further studies could provide data to clarify these factors and guide strategies to improve access. Optimizing surgical scheduling and resource allocation within the public sector could represent a cost-effective strategy to address waiting lists and ensure equitable service delivery. In response to rising cataract surgery waiting lists, the national health service introduced a time-limited out-of-hours operating list, with staff remunerated per case (37). The initiative aimed to reduce delays and meet the six-month target outlined in the patient charter, but also introduced additional direct costs. Although not intended as a permanent funding model, it can be viewed economically as a targeted investment to address a backlog and restore timely service delivery. A cost–utility analysis may help assess the value of this intervention by comparing its costs to the quality-adjusted-life-years (QALYs) gained through earlier access to surgery. Such analysis may inform the design and justification of future time-limited measures to manage elective surgery backlogs in a cost-effective and patient-centered manner. However, with the ageing of the Maltese population and the likely growth in demand for surgical eye care, sustainable workforce planning will be equally important. This includes ensuring adequate numbers of trained ophthalmologists, anesthetic support, and theater staff to meet routine demand, thereby reducing the need for *ad hoc* backlog-clearing interventions. Further longitudinal data are required to monitor the growing demand on the healthcare system and to inform better public healthcare planning and resource allocation, including projections of the ophthalmic workforce required to meet future cataract surgical needs.

The proportion of unmet refractive needs requires further research on the barriers toward successful refractive management, including health literacy, compliance to eye checks and difficulty or delays in accessing eye care checks. URE, although associated with relatively low direct treatment costs (€1.2 million), contributes substantially to productivity losses (€8.2 million). These findings support the potential value of public awareness campaigns promoting routine eye refraction among working-age adults and the expansion of public-sector optometric services, which currently comprise only four of 30 practicing optometrists. Screening for refractive error is cost-effective in other contexts; for example, a community-based program in Hong Kong estimated the cost to prevent one case of distance visual impairment at between HK$1921 (US$246) and HK$3715 (US$476) from the funder’s perspective (13). Globally, the estimated costs of establishing and operating refractive care facilities are significantly lower than the productivity losses associated with untreated URE (45). Expanding the role of optometrists in emergency eye care, cataract and glaucoma referrals, and optical coherence tomography services has also been shown elsewhere to reduce hospital appointment volumes and associated costs (46, 47).

Screening for DR among individuals with no more than mild non-proliferative changes was estimated to cost approximately €450,000 annually, based on current service prices. Conversely, the cost of managing more advanced DR exceeded €2.4 million annually, even when surgical and laser interventions were excluded. This difference indicated that expanded screening may be economically favorable, since it improves early detection and may cut down on costs related to more deteriorated DR and DR-related VI. Other population-based studies have recently shown the added utility of using artificial intelligence in DR screening, typically at under US $20,000 per QALY gained (48, 49). A health economic analysis of the current screening protocol for DR (as obtained via personal communication with Micallef B., 2025), could estimate its direct, indirect, and capital costs in relation to its effectiveness in reducing the economic burden of VI and blindness caused by DR. A cost-savings or cost–utility approach may be most appropriate, since expressing both costs and benefits in monetary terms is rarely feasible in this context. The evolving costs of VI due to DR would ideally be derived from real-time data sourced from a VI registry or electronic health records. Previous findings from TMES indicate that individuals with diabetes are more likely to experience cataract-related VI (16). Incorporating cataract and refractive error screening for these individuals within the DR program may be cost-effective; for example, biennial eye evaluations, which can detect other eye conditions, including cataracts and refractive errors, were more cost-effective than annual evaluations (50).

The potential public sector adoption of agents such as aflibercept, an intravitreal drug used for both DME and ARMD, which allows for longer injection intervals than the presently available bevacizumab and ranibizumab, may warrant a formal health economic analysis based on local outcomes. Such an evaluation could take the form of a cost–utility analysis using QALYs, which would enable comparison with other healthcare interventions and alignment with international thresholds for adoption, such as those applied by the National Institute for Health and Care Excellence in the UK (approximately £20,000 per QALY) (51).

Glaucoma remains underdiagnosed, with the majority of cases or suspects not currently being addressed (Table 8 shows 3,575 meeting their needs while 15,705 not meeting their needs). This proportion has persisted since the 1989 study by Cachia et al. (52). The reason for this ongoing gap needs further evaluation, especially regarding health literacy and willingness to attend eye checks among individuals with unknown glaucoma.

Expansion of vision screening programs for cataract, glaucoma, DR, and ARMD targeting older adult individuals has been introduced (53, 54), but their cost-effectiveness remains to be evaluated. Future work could explore the optimal starting age, the role of practice nurses in detecting glaucoma and other eye conditions, and the use of technology (such as artificial intelligence) to support a Malta-wide primary care-focused program.

## Conclusion

This study provides the first comprehensive estimate of the economic burden of VI in Malta in an older adult population, with productivity losses reaching €16.0 million annually, direct medical costs exceeding €53.0 million (mainly from cataract and URE), and URE carrying the highest YLD rate among the VI causes (2452.7 YLDs per 100,000). The findings underscore the importance of further evaluating the cost-effectiveness of current and novel prevention and treatment strategies in order to minimize such costs. They also highlight the need for improved health system data, integrated electronic records, and health economic evaluations of targeted interventions such as expanded diabetic retinopathy screening, cataract backlog initiatives, and optometric service provision and country-wide eye health screening initiatives for all ages. Future cost-effectiveness analyses will be critical to guide resource allocation and inform sustainable public eye health policy.

## Data Availability

The datasets presented in this article are not readily available because they contain sensitive health information and are protected under the conditions of our ethics approval. Requests to access the datasets should be directed to the corresponding author at david.agius@um.edu.mt, and will be considered on a case-by-case basis following the required ethical clearance.
