# Supplementary material for: Estimating the costs and quality of life impact of vision loss in the population aged 50-80 years in Malta: evidence from The Malta Eye Study
Source: Front Public Health. 2025 Dec 5;13:1706208. doi: 10.3389/fpubh.2025.1706208 (PMC12714930; doi:10.3389/fpubh.2025.1706208)
Supplement: Supplementary file 1 [file Table_1.DOCX]

| **Supplemental Table 1: Prevalence of Visual Impairment by Cause, Age Group and Severity of Visual Impairment in TMES and Extrapolated Population Numbers** | | | | | | | | |
| --- | --- | --- | --- | --- | --- | --- | --- | --- |
| **Cause of Visual Impairment** | **Age Group** | **Severity of Visual Impairment** | **Prevalence** | | | **Extrapolated Number in Population** | | |
|  |  |  | **%** | **L95%CI** | **U95%CI** | **n** | **L95%CI** | **U95%CI** |
| **Uncorrected Refractive Error** | **50-64** | **MSVI** | 0.7% | 0.3% | 1.5% | 674 | 271 | 1383 |
|  |  | **Blindness** | 0.0% | 0.0% | 0.4% | 0 | 0 | 354 |
|  | **65-80** | **MSVI** | 0.6% | 0.2% | 1.4% | 463 | 150 | 1076 |
|  |  | **Blindness** | 0.0% | 0.0% | 0.4% | 0 | 0 | 341 |
| **Amblyopia** | **50-64** | **MSVI** | 0.2% | 0.0% | 0.8% | 192 | 23 | 693 |
|  |  | **Blindness** | 0.0% | 0.0% | 0.4% | 0 | 0 | 354 |
|  | **65-80** | **MSVI** | 0.4% | 0.1% | 1.0% | 278 | 57 | 809 |
|  |  | **Blindness** | 0.0% | 0.0% | 0.4% | 0 | 0 | 341 |
| **Cataract** | **50-64** | **MSVI** | 0.0% | 0.0% | 0.4% | 0 | 0 | 354 |
|  |  | **Blindness** | 0.1% | 0.0% | 0.6% | 96 | 2 | 535 |
|  | **65-80** | **MSVI** | 0.5% | 0.1% | 1.2% | 370 | 101 | 944 |
|  |  | **Blindness** | 0.1% | 0.0% | 0.7% | 93 | 2 | 514 |
| **Diabetic Retinopathy** | **50-64** | **MSVI** | 0.0% | 0.0% | 0.4% | 0 | 0 | 354 |
|  |  | **Blindness** | 0.0% | 0.0% | 0.4% | 0 | 0 | 354 |
|  | **65-80** | **MSVI** | 0.2% | 0.0% | 0.8% | 185 | 22 | 667 |
|  |  | **Blindness** | 0.1% | 0.0% | 0.7% | 93 | 2 | 514 |
| **Age-Related Macular Degeneration** | **50-64** | **MSVI** | 0.0% | 0.0% | 0.4% | 0 | 0 | 354 |
|  |  | **Blindness** | 0.0% | 0.0% | 0.4% | 0 | 0 | 354 |
|  | **65-80** | **MSVI** | 0.1% | 0.0% | 0.7% | 93 | 2 | 514 |
|  |  | **Blindness** | 0.1% | 0.0% | 0.7% | 93 | 2 | 514 |
| **Glaucoma** | **50-64** | **MSVI** | 0.0% | 0.0% | 0.4% | 0 | 0 | 354 |
|  |  | **Blindness** | 0.0% | 0.0% | 0.4% | 0 | 0 | 354 |
|  | **65-80** | **MSVI** | 0.0% | 0.0% | 0.4% | 0 | 0 | 341 |
|  |  | **Blindness** | 0.1% | 0.0% | 0.7% | 93 | 2 | 514 |
| **Pathological Myopia** | **50-64** | **MSVI** | 0.0% | 0.0% | 0.4% | 0 | 0 | 354 |
|  |  | **Blindness** | 0.0% | 0.0% | 0.4% | 0 | 0 | 354 |
|  | **65-80** | **MSVI** | 0.4% | 0.1% | 1.0% | 278 | 57 | 809 |
|  |  | **Blindness** | 0.0% | 0.0% | 0.4% | 0 | 0 | 341 |

| **Supplemental Table 2: Annual estimated productivity losses arising from individuals with blindness and MSVI by cause aged 50-80 in Malta** | | | | | | | | | | |
| --- | --- | --- | --- | --- | --- | --- | --- | --- | --- | --- |
| **Cause** | **Visual Impairment Group** | **Age Group of Impaired Individuals** | **Individual /Carer Losses** | **Gross National Income Per capita Calculation** | | | **National Minimum Wage Calculation** | | |  |
|  |  |  |  | **Loss (€)** | **L 95% CI (€)** | **U 95% CI (€)** | **Loss (€)** | **L 95% CI (€)** | **U 95% CI (€)** |  |
| **Uncorrected Refractive Error** | **Blindness** | **50-64** | Individual | 0 | 0 | 11,172,067 | 0 | 0 | 3,934,371 |  |
|  |  |  | Carer | 0 | 0 | 1,117,207 | 0 | 0 | 393,437 |  |
|  |  | **65-80** | Carer | 0 | 0 | 1,074,198 | 0 | 0 | 378,291 |  |
|  |  | **Total** | **Total** | **0** | **0** | **13,363,472** | **0** | **0** | **4,706,099** |  |
|  | **Moderate Severe Visual Impairment** | **50-64** | Individual | 6,372,533 | 2,566,453 | 13,077,951 | 2,244,160 | 903,806 | 4,605,549 |  |
|  |  |  | Carer | 1,062,089 | 427,742 | 2,179,659 | 374,027 | 150,634 | 767,592 |  |
|  |  | **65-80** | Carer | 729,582 | 237,226 | 1,695,915 | 256,931 | 83,542 | 597,236 |  |
|  |  | **Total** | **Total** | **8,164,204** | **3,231,421** | **16,953,525** | **2,875,117** | **1,137,982** | **5,970,377** |  |
|  | **Total** | **Total** | **Total** | **8,164,204** | **3,231,421** | **30,316,997** | **2,875,117** | **1,137,982** | **10,676,475** |  |
| **Amblyopia** | **Blindness** | **50-64** | Individual | 0 | 0 | 11,172,067 | 0 | 0 | 3,934,371 |  |
|  |  |  | Carer | 0 | 0 | 1,117,207 | 0 | 0 | 393,437 |  |
|  |  | **65-80** | Carer | 0 | 0 | 1,074,198 | 0 | 0 | 378,291 |  |
|  |  | **Total** | **Total** | **0** | **0** | **13,363,472** | **0** | **0** | **4,706,099** |  |
|  | **Moderate Severe Visual Impairment** | **50-64** | Individual | 1,820,724 | 220,587 | 6,558,788 | 641,189 | 77,682 | 2,309,752 |  |
|  |  |  | Carer | 303,454 | 36,765 | 1,093,131 | 106,865 | 12,947 | 384,959 |  |
|  |  | **65-80** | Carer | 437,749 | 90,348 | 1,274,945 | 154,158 | 31,817 | 448,986 |  |
|  |  | **Total** | **Total** | **2,561,927** | **347,700** | **8,926,864** | **902,212** | **122,446** | **3,143,697** |  |
|  | **Total** | **Total** | **Total** | **2,561,927** | **347,700** | **22,290,336** | **902,212** | **122,446** | **7,849,795** |  |
| **Cataract** | **Blindness** | **50-64** | Individual | 3,034,539 | 76,827 | 16,866,227 | 1,068,648 | 27,055 | 5,939,634 |  |
|  |  |  | Carer | 303,454 | 7,683 | 1,686,623 | 106,865 | 2,706 | 593,963 |  |
|  |  | **65-80** | Carer | 291,833 | 7,388 | 1,621,612 | 102,772 | 2,602 | 571,069 |  |
|  |  | **Total** | **Total** | **3,629,826** | **91,898** | **20,174,462** | **1,278,285** | **32,363** | **7,104,666** |  |
|  | **Moderate Severe Visual Impairment** | **50-64** | Individual | 0 | 0 | 3,351,620 | 0 | 0 | 1,180,311 |  |
|  |  |  | Carer | 0 | 0 | 558,603 | 0 | 0 | 196,719 |  |
|  |  | **65-80** | Carer | 583,666 | 159,209 | 1,488,924 | 205,544 | 56,067 | 524,341 |  |
|  |  | **Total** | **Total** | **583,666** | **159,209** | **5,399,147** | **205,544** | **56,067** | **1,901,371** |  |
|  | **Total** | **Total** | **Total** | **4,213,492** | **251,107** | **25,573,609** | **1,483,829** | **88,430** | **9,006,037** |  |
| **Diabetic Retinopathy** | **Blindness** | **50-64** | Individual | 0 | 0 | 11,172,067 | 0 | 0 | 3,934,371 |  |
|  |  |  | Carer | 0 | 0 | 1,117,207 | 0 | 0 | 393,437 |  |
|  |  | **65-80** | Carer | 291,833 | 7,388 | 1,621,612 | 102,772 | 2,602 | 571,069 |  |
|  |  | **Total** | **Total** | **291,833** | **7,388** | **13,910,886** | **102,772** | **2,602** | **4,898,877** |  |
|  | **Moderate Severe Visual Impairment** | **50-64** | Individual | 0 | 0 | 3,351,620 | 0 | 0 | 1,180,311 |  |
|  |  |  | Carer | 0 | 0 | 558,603 | 0 | 0 | 196,719 |  |
|  |  | **65-80** | Carer | 291,833 | 35,358 | 1,050,958 | 102,772 | 12,452 | 370,107 |  |
|  |  | **Total** | **Total** | **291,833** | **35,358** | **4,961,181** | **102,772** | **12,452** | **1,747,136** |  |
|  | **Total** | **Total** | **Total** | **583,666** | **42,747** | **18,872,068** | **205,544** | **15,054** | **6,646,013** |  |
| **Age-Related Macular Degeneration** | **Blindness** | **50-64** | Individual | 0 | 0 | 11,172,067 | 0 | 0 | 3,934,371 |  |
|  |  |  | Carer | 0 | 0 | 1,117,207 | 0 | 0 | 393,437 |  |
|  |  | **65-80** | Carer | 291,833 | 7,388 | 1,621,612 | 102,772 | 2,602 | 571,069 |  |
|  |  | **Total** | **Total** | **291,833** | **7,388** | **13,910,886** | **102,772** | **2,602** | **4,898,877** |  |
|  | **Moderate Severe Visual Impairment** | **50-64** | Individual | 0 | 0 | 3,351,620 | 0 | 0 | 1,180,311 |  |
|  |  |  | Carer | 0 | 0 | 558,603 | 0 | 0 | 196,719 |  |
|  |  | **65-80** | Carer | 145,916 | 3,694 | 810,806 | 51,386 | 1,301 | 285,535 |  |
|  |  | **Total** | **Total** | **145,916** | **3,694** | **4,721,030** | **51,386** | **1,301** | **1,662,564** |  |
|  | **Total** | **Total** | **Total** | **437,749** | **11,083** | **18,631,916** | **154,158** | **3,903** | **6,561,441** |  |
| **Glaucoma** | **Blindness** | **50-64** | Individual | 0 | 0 | 11,172,067 | 0 | 0 | 3,934,371 |  |
|  |  |  | Carer | 0 | 0 | 1,117,207 | 0 | 0 | 393,437 |  |
|  |  | **65-80** | Carer | 291,833 | 7,388 | 1,621,612 | 102,772 | 2,602 | 571,069 |  |
|  |  | **Total** | **Total** | **291,833** | **7,388** | **13,910,886** | **102,772** | **2,602** | **4,898,877** |  |
|  | **Moderate Severe Visual Impairment** | **50-64** | Individual | 0 | 0 | 3,351,620 | 0 | 0 | 1,180,311 |  |
|  |  |  | Carer | 0 | 0 | 558,603 | 0 | 0 | 196,719 |  |
|  |  | **65-80** | Carer | 0 | 0 | 537,099 | 0 | 0 | 189,145 |  |
|  |  | **Total** | **Total** | **0** | **0** | **4,447,323** | **0** | **0** | **1,566,175** |  |
|  | **Total** | **Total** | **Total** | **291,833** | **7,388** | **18,358,209** | **102,772** | **2,602** | **6,465,052** |  |
| **Pathological Myopia** | **Blindness** | **50-64** | Individual | 0 | 0 | 11,172,067 | 0 | 0 | 3,934,371 |  |
|  |  |  | Carer | 0 | 0 | 1,117,207 | 0 | 0 | 393,437 |  |
|  |  | **65-80** | Carer | 0 | 0 | 1,074,198 | 0 | 0 | 378,291 |  |
|  |  | **Total** | **Total** | **0** | **0** | **13,363,472** | **0** | **0** | **4,706,099** |  |
|  | **Moderate Severe Visual Impairment** | **50-64** | Individual | 0 | 0 | 3,351,620 | 0 | 0 | 1,180,311 |  |
|  |  |  | Carer | 0 | 0 | 558,603 | 0 | 0 | 196,719 |  |
|  |  | **65-80** | Carer | 437,749 | 90,348 | 1,274,945 | 154,158 | 31,817 | 448,986 |  |
|  |  | **Total** | **Total** | **437,749** | **90,348** | **5,185,169** | **154,158** | **31,817** | **1,826,016** |  |
|  | **Total** | **Total** | **Total** | **437,749** | **90,348** | **18,548,641** | **154,158** | **31,817** | **6,532,115** |  |

| **Supplemental Table 3: Mean Disability Weights (DW) with 95% Confidence Intervals, Prevalence, and Prevalence-Based Years Lived with Disability (YLD) for the Highest-Burden Visual Impairment (VI) Types by Age Group in the Census-Adjusted TMES Population** | | | | | | | | | | | | | |
| --- | --- | --- | --- | --- | --- | --- | --- | --- | --- | --- | --- | --- | --- |
| **Visual Impairment Group** | **Age Group** | **Mean DW** | | | **Prevalence (%)** | | | **Absolute YLD number** | | | **YLD rate per 100,000** | | |
|  |  | DW | L95%CI | U95%CI | Prevalence | L95%CI | U95%CI | **YLD** | L95%CI | U95%CI | **YLD** | L95%CI | U95%CI |
| **Mild Unilateral VI** | 50-59 | 0.18 | 0.16 | 0.21 | 7.9% | 5.9% | 10.3% | **849.4** | 552.2 | 1251.7 | **1440.1** | 936.2 | 2122.1 |
|  | 60-69 | 0.23 | 0.21 | 0.25 | 10.0% | 7.7% | 12.6% | **1375.9** | 980.7 | 1878.9 | **2303.7** | 1642.0 | 3145.9 |
|  | 70-80 | 0.23 | 0.22 | 0.25 | 13.4% | 10.7% | 16.7% | **1576.8** | 1152.2 | 2104.9 | **3150.6** | 2302.3 | 4205.8 |
| **Uncorrected Refractive Error VI** | 50-59 | 0.18 | 0.16 | 0.20 | 11.2% | 8.8% | 13.9% | **1177.2** | 813.0 | 1648.4 | **1995.8** | 1378.4 | 2794.6 |
|  | 60-69 | 0.22 | 0.20 | 0.24 | 12.5% | 10.0% | 15.3% | **1633.5** | 1197.2 | 2175.1 | **2735.0** | 2004.5 | 3641.8 |
|  | 70-80 | 0.22 | 0.20 | 0.23 | 13.2% | 10.5% | 16.4% | **1424.5** | 1026.3 | 1923.9 | **2846.3** | 2050.7 | 3844.1 |
| **Cataract VI** | 50-59 | 0.31 | 0.00 | 1.15 | 0.8% | 0.3% | 1.9% | **147.1** | 0.0 | 1264.1 | **249.4** | 0.0 | 2143.2 |
|  | 60-69 | 0.28 | 0.22 | 0.33 | 1.7% | 0.9% | 3.1% | **287.9** | 115.2 | 614.6 | **482.0** | 192.8 | 1029.1 |
|  | 70-80 | 0.27 | 0.24 | 0.29 | 9.6% | 7.3% | 12.5% | **1279.1** | 872.4 | 1812.7 | **2555.8** | 1743.0 | 3622.0 |
| **Amblyopia VI** | 50-59 | 0.17 | 0.14 | 0.19 | 3.8% | 2.5% | 5.7% | **374.0** | 201.1 | 642.7 | **634.0** | 340.9 | 1089.5 |
|  | 60-69 | 0.21 | 0.18 | 0.23 | 6.2% | 4.4% | 8.3% | **759.2** | 468.0 | 1170.0 | **1271.2** | 783.6 | 1959.0 |
|  | 70-80 | 0.23 | 0.19 | 0.26 | 5.1% | 3.4% | 7.3% | **584.1** | 327.9 | 970.1 | **1167.1** | 655.1 | 1938.4 |
| Negative lower confidence bounds for the disability weight of bilateral blindness, resulting from small sample size, were truncated to 0 as values below 0 are not meaningful. | | | | | | | | | | | | | |
